# Medical Legislation

**Published:** 1881-02

**Authors:** 


### Editorial.

---

MEDICAL LEGISLATION.—The time has come again, when the legislative bodies of most of the states are in session and ready to legislate on all conceivable subjects.

During the last few years there has been an increasing disposition on the part of these legislative bodies to enact laws for the protection of the people both against the spread of contagious and infectious diseases, and the impositions of unqualified medical practitioners. Both these objects are of very great importance to the people, and are, therefore, worthy of the most careful consideration, especially by all intelligent physicians. In regard to the enactment of laws for the protection of the people against the impositions and dangers of unqualified *Doctors;* or, in other words, laws to secure for the people the services of a thoroughly educated medical profession, there are now, and always have been, practical difficulties not easily overcome.

These arise in part from a want of knowledge on the part of the people, or their representatives in the legislatures, concerning the nature of medical studies and the means necessary to prosecute them successfully; and in part from the difficulty of establishing and maintaining proper and efficient tribunals in each State for exacting impartially the proper standard of attainments from all those who propose to enter upon the practice of medicine.

An adequate knowledge of the first, would show the members of every legislative body: first, that no person could be properly prepared to commence the study of any department of medical science until his mind had been disciplined and stored by a thorough study of all the ordinary branches of an English education, including mathematics, physics, and the natural sciences. Second, that the very nature of most of the branches of medical science, such as anatomy, physiology, chemistry, materia medica, pathology, practical medicine, surgery, midwifery, etc., absolutely require for their practical study the use of material, apparatus, and means of illustration, that can be found no where except in medical colleges, and hospitals for the sick. So large a proportion of the whole field of medical knowledge requires for its proper practical acquisition the means furnished only by such institutions, that, at least, one-half of the whole period allotted to medical studies should be spent in them. Third, that a medical college, to fulfill the purposes for which such institutions are needed, must have a sufficient supply of anatomical material; sufficient chemical, physiological, and pathological laboratories and apparatus; adequate cabinets or museums of materia medica, pathological specimens, comparative anatomy, etc.; and such intimate connection with permanently established hospitals and dispensaries for the sick as will afford ample opportunity to study disease at the bedside; in other words, that a proper medical college for the education of young men to practice medicine, is something more than the organization of six or eight doctors into a college faculty under some special charter or general incorporation law, with an annual term of from sixteen to twenty weeks of simple didactic instruction, with the power to grant *diplomas* to whoever they please at the end of it.

These three propositions, so plain and familiar to every intelligent and thoughtful medical man, but altogether obscure to the ordinary, non-professional members of our legislature, show very clearly that no *law* can be devised which will practically insure to the people the services of a body of well qualified and reliable physicians and surgeons, without including in its provisions: first, a proper standard of preliminary education to be determined before entering upon the study of medicine; second, the minimum length of time that must be devoted directly to the study of medicine, and how much of that time shall be spent in attendance on a proper medical college; and third, what shall constitute a medical college to be recognized under the law.

Yet most of the laws that have been enacted in the several

States, during the last twenty years, for the regulation of the practice of medicine have wholly failed to regard any of these fundamental propositions. The law of this State passed in 1877, for regulating the practice of medicine and establishing a State Board of Health, does not touch either of these topics; but simply requires the applicant for license to practice in the State, to present and verify the rightful possession of a *diploma* from some "chartered or legally established medical college in good standing," or undergo an examination in the several branches of medicine by the Board of Health.

The law does not say whether the college issuing the "*diploma*" must have any adequate means for imparting medical instruction, or whether it must require the attendance of the student one, two or three terms, or whether the terms shall be one or six months long; but only that it be legally established and in good standing. But when it is remembered that any five or more physicians or even non-professional men can "legally" establish a medical college under the general incorporation laws of most of the states, and that the words, "good standing," are altogether too vague for any practical use, it will be apparent that the law really amounts to nothing more than one of registration, without any power to exact a given standard of education.

So, too, the power given to the State Board to examine and license those who have no diploma, is equally indefinite. It does not restrict the Board to an examination of those only who have faithfully devoted three years or more to the study of medicine after having had a proper preliminary education, and who have spent at least half of that time in some properly organized medical college and hospital; but leaves it entirely at liberty to examine any applicant they please, whether he has studied six months or six years, or has ever seen the inside of a medical college or not. It is needless to add that the value of such a law in securing to the people the services of a thoroughly qualified medical profession, must be very small; and even the little that it can accomplish depends entirely on the character and faithfulness of the members of the State Board. And this leads to some remarks in regard to what we have mentioned as the second source of difficulty in the framing of efficient laws for maintaining a proper standard of medical education, namely, the creation and perpetuation of an efficient State board or executive medical body, whose duty should be to see that the laws relating to the proper qualification of medical practitioners were faithfully observed and executed. All will agree that such a board should be composed mainly, if not exclusively, of medical men of the highest degree of probity and learning, and its powers and duties should be clearly defined. If the law regulating the practice of medicine in this State should be so amended that after a given date (say January, 1883), no one should be permitted to enter upon the practice of medicine within its limits except those who should present to the State Board a diploma from some medical college which required for admission to its halls such a preliminary education as we have already indicated, and three full years of medical study; attendance on three annual courses of college instruction of not less than six months each, and regular attendance on clinical instruction in a general hospital for the sick, at least, one collegiate year, before granting the diploma; then the question as to how and by whom the board should be appointed, would be of less consequence simply because the duties of the board under such a law would be chiefly clerical. To verify the genuineness of the diploma and the rightful possession of it, and the true status of the college granting it, would constitute their chief duty in this direction. For the execution of such a law, our present State Board of Health, or any other board similiarly constituted and appointed, would not be objectionable.

But if the board is to retain the power to *examine* and *license* any who have not received a diploma from some recognized medical college, it should be compelled to limit such examinations to those applicants who had complied with all the requirements necessary for being admitted to the final examination for a diploma in a college of recognized standing.

If these alterations were made in our State law, it would at once fix a minimum standard for the education of the profession of a fair and honorable character, make a reliable guide for the action of the State Board, and it would compel a most desirable and general advance in the standard of medical college requirements and instruction, and yet it would inflict no injury on any medical school in our own State. To effect the objects here proposed it would only be necessary to amend the present law for regulating the practice of medicine, passed in 1877, by inserting the following after the words "good standing," in section 3: [but the diploma of no medical institution shall be received that does not enforce the requirement of a good academic or high-school education for the admission of students; and exact the study of medicine at least three years, direct attendance on the college instruction at least three annual terms of not less than six months each, and on the clinical instruction in a general hospital at least one college term, and a full examination in all the branches taught, as conditions for graduation.] And, in the same section, after the words "examined by the Board," insert the following: [but no person shall be thus examined by the Board unless he has fully complied with all the educational requirements that would be necessary to entitle him to an examination for a diploma by a recognized medical college.] D.

